# A New Crystal Engineering Technique for Dissolution Enhancement of Poorly Soluble Drugs Combining Quasi-emulsion and Crystallo-co Agglomeration Methods

**DOI:** 10.22037/ijpr.2020.1101094

**Published:** 2020

**Authors:** Rana R. Makar, Randa Latif, Ehab A. Hosni, Omaima N. El Gazayerly

**Affiliations:** a *Department of Pharmaceutics, Faculty of Pharmacy, Ahram Canadian University, Egypt. *; b *Department of Pharmaceutics and Industrial Pharmacy, Faculty of Pharmacy, Cairo University, Egypt.*; c *Department of Pharmaceutics, Faculty of Pharmacy, Egyptian Russian University, Egypt.*

**Keywords:** Spherical crystallization, Quasi-emulsion, Crystallo-co-agglomeration, Dissolution enhancement, Poorly soluble drugs

## Abstract

A target of best dissolution improvement of poorly soluble drugs is a necessity for the success of formulation in industry. The present work describes the preparation, optimization, and evaluation of a new spherical agglomeration technique for glimepiride as a model of poorly soluble drugs. It involved the emulsification of a drug solution containing a dispersed carrier that tailors the crystal habit of the drug to a perfect spherical geometry, in a poor solvent containing a hydrophilic polymer which imparts sphericity and strength to the formed agglomerates. The FTIR peaks of optimized product did not show any sign of chemical interaction between the drug and adsorbed carrier. The DSC and X ray diffractogram showed a peak characteristic of spherical agglomerates with much less intensity than that of glimepiride. The dissolution t_1/2 _of the drug slightly decreased from 381 min to 334 min in plain agglomerates. Introducing polymers in the aqueous phase of emulsion led to an improvement in the dissolution, reflected in t_1/2 _ranging from 118 to 231 min. Agglomerates prepared with Starlac/PVP demonstrated the most optimum physicochemical characteristics being spherical, with the best flowability and packability parameters. The t_1/2_ was as short as 19 min. The new carrier/polymer system offered a synergistic combination that highly contributed in dissolution enhancement of glimepiride. The spheronization and amorphisation offered by the new technique could account for such improvement.

## Introduction

The development of new methods for enhancing the dissolution of poorly soluble drugs presents a great challenge in pharmaceutical industry (1, 2).

The dissolution of drugs belonging to BCSII class was thought to be the rate-limiting step for their absorption and bioavailability (3). Solid dispersion is one of the useful techniques that greatly treat the poor dissolution rate of these drugs (4). One of the reasons for the wide spreading of the technique is the use of amorphous starch based carbohydrate polymers like pregelatinized starch, Glycolys^®^, Starlac, Pearlitol flash and others (5- 9). These carriers increase the wettability and dispersability of drugs and also inhibit the precipitation of drugs when solid dispersion is dissolved in water (10).

Recent researches have illustrated the possibility of tailoring the crystal shape or morphology, (size and properties of drug crystals, which could play a key role in drug development and formulation (11-14).

Spherical agglomeration was reported among the most efficient techniques that could improve the micromeritics as well as the dissolution of poorly crystalline drugs (15-18). The technique can directly transform the fine crystals produced in the crystallization vessel into spherically shaped particles. A general strategy exists for most spherical agglomeration techniques. This could be summarized in three steps; selection of the crystallization method to precipitate crystals from the solution; choice of the wetting agent that will be immiscible with the solvent of crystallization and is commonly named as bridging liquid; and finally, hardening of the agglomerates (19).

Quasi-emulsion solvent diffusion was cited among the techniques which were usually applied for the preparation of microspheres (20). The technique involved a stronger interaction between a drug and a good solvent, along with a weaker interaction between good and poor solvents. Hence, the drug solution (dispersed in the poor solvent) produced quasi-emulsion droplets, even though the solvents were miscible. Then the good solvent diffused gradually out of the emulsion droplets into the outer poor solvent phase. The counter-diffusion of the poor solvent into the droplets induced crystallization of the drug within the droplets in the form of spherical agglomerates (11, 21 and 22).

Another modification of the agglomeration technique is the inclusion of special excipients along with the drug during the simple crystallization process; the so-called crystallo-co-agglomeration technique. This method enables the design of the agglomerates of poorly compressible drugs or the agglomeration of two drugs together. In this case the selection of a solvent system will largely depend on the physicochemical properties of the drug and excipients used (23- 25).

To our knowledge, literature lacks any data about the preparation of a combined form from the two aforementioned agglomeration techniques. Thus, the goal of the present work was to investigate the applicability of starch based carbohydrate polymers as promising carriers in a novel spherical crystallisation procedure combining features of quasi-emulsion and crystallo-co-agglomeration.

The study was then directed towards the optimization of different studied variables including type and percent of added polymer on the success of spherical crystallisation. The final aim was to reach the best agglomeration and spheronization of particles which assist their amorphisation and dissolution enhancement.

## Experimental

*Materials*

Glimepiride was kindly supplied by Sedico Pharmaceuticals, Giza, Egypt. Sodium Lauryl Sulphate was purchased from El-Nasr Pharmaceutical Chemicals Co., Cairo, Egypt. Pregelatinized starch (PreGelSt) was supplied from Colorcon Limited, UK. Ac-Di-Sol (Crosscarmellose sodium) was purchased from E. Merck, Germany. Crosspovidone XL (CP) was purchased from FMC Corporation, Philadelphia, USA.

Glycolys^®^ (sodium carboxymethyl starch), Starlac (lactose and maize starch) and Pearlitol flash (mannitol and maize starch) were a kind gift from Roquette, France.

Gelucire 44/14 and Gelucire 50/13 were supplied from Gattefosé, France.

Colloidal Silicon dioxide (Aerosil 200) hydrophilic was supplied from Degussa, USA. 

Polyvinylpyrrolidone (PVP K30) was purchased from Fluka, Switzerland.

Low substituted Hydroxypropylcellulose (L-HPC) was got from Shin-Etsu Chemical Co., Ltd Tokyo, Japan. Polyvinyl alcohol (PVA) was obtained from Sigma –Aldrich, Germany.

Dimethylformamide (DMF), Dichloromethane (Methylene chloride/DCM), Chloroform (CHCl_3_), Carbon tetra chloride (CCL_4_), Toluene and Benzene were supplied from Fine-Chem limited, Mumbai, India.

*Optimization of spherical agglomerates (SA)*

*Preliminary trials*

Table 1 shows the tested variables during the optimization of spherical agglomeration process. Glimepiride was dissolved either in a good solvent (DMF or DCM) or a mixture of solvents (DMF and benzene) in a small beaker at 40 °C so as to form a saturated solution. The poor solvent (distilled water) was added to precipitate the drug. The mixture was then agitated using a three-blade mechanical stirrer, and the bridging liquid CHCl_3_, Toluene, CCl_4 _or Benzene was added drop-wise with agitation. 

Different agitation speeds were tried; 200 rpm, 500 rpm, and 900 rpm at 25 ºC and 40 ºC. After agitation for 10 min, the prepared agglomerates were collected, washed with distilled water, filtered (through Whatman filter paper number one) and placed at 45 °C for drying in a hot air oven for 24 h. The dried agglomerates were tested for the absence of traces of organic solvent using FT-IR spectroscopy and then stored in tightly closed containers in a desiccator for further investigation.

*Addition of Aerosil in the drug solution*

After obtaining the optimum ratios of the drug: good solvent: poor solvent: bridging liquid, the same experiment was repeated with the addition of Aerosil 200 to the drug solution with continuous agitation to keep the suspension uniformly dispersed (Table 2).

*Addition of hydrophilic polymers in the aqueous phase*

The same experiment was repeated with the addition of different hydrophilic polymers (PVA, HPC and PVP K 30) to SA, namely SAPVA, SAHPC and SAPVP respectively in an aqueous solution (Table 2).

Each tested hydrophilic polymer was dissolved in water until a saturated solution was formed at room temperature, then the prepared aqueous solution was added to the drug solution with continuous agitation. CCl_4_ was added drop-wise.

*Addition of different carriers in the drug solution*

The same experiment was repeated with the addition of a carrier to the drug solution with continuous agitation to keep it uniformly dispersed (Table 2).

Six carriers were tested viz: PreGelSt, Ac-Di-Sol, Glycolys^®^, CP, Starlac and Pearlitol flash. Six formulae were respectively prepared, namely, SAps, SAac, SAgl, SAcp, SAst, and SApf.

*Physicochemical evaluation of the prepared spherical agglomerates*

*Determination of the yield percent and drug content*

Each system was weighed after complete drying to determine the yield percent according to the following formula

$\frac{\mathrm{Actual weight}}{\mathrm{Theoretical weight}}\times 100(26)$.

The drug content was determined by dispersing a specified weight from each system in 50 mL DCM with the aid of magnetic stirring for 15 min. The solutions were filtered and the absorbance of glimepiride was measured spectrophotometrically at λ max 228 nm (Spectrophotometer: Shimadzu, UV-2401 PC, Australia) after doing the appropriate dilution. The results were the mean of three determinations.

*In-vitro* *drug dissolution*

The dissolution profile of the drug in prepared spherical agglomerates (SA) with or without different carriers and/or hydrophilic polymers was determined using USP dissolution tester (Hanson Research, 64-705-045, USA). An accurately weighed amount of each system equivalent to 3 mg of the drug was placed in a rotating basket (at 100 rpm) covered by a standardized mesh packet. The dissolution was carried out at 37 °C in 900 mL 0.5% aqueous solution of SLS. Two ml samples were withdrawn at different time intervals and replaced with fresh media. The absorbance of the samples was measured spectrophotometrically at λ max 228 nm. The results were mean of three determinations.

*Kinetic analysis of the dissolution data*

The data obtained from dissolution experiments were treated statistically according to linear regression analysis. The data were fitted to zero order, first order and Higuchi diffusion model. Kinetic treatment of the data was then performed for the order of the best fit.

Equation for zero order: $C=C_{{}^{\circ}}-K_{{}^{\circ}}t$

Equation for first order:$\log C=\log C_{{}^{\circ}}-\mathrm{Kt}/2.303$

Simplified equation for Higuchi diffusion model:$Q=K\times t_{1/2}$

K was calculated from the slope of the straight line and t_1/2 _deduced by replacement in the respective equation of the best fit.

*Micromeritics characterization of optimized spherical agglomerates*

Systems that showed promising dissolution profiles were chosen for further investigation.

The flowability of pure drug and spherical agglomerate samples was assessed by the determination of the angle of repose (27, 28), Hausner’s ratio and Carr’s index (29, 30).

*Angle of repose*

The angle of repose was determined by the fixed funnel method. The mean of three determinations was calculated. 

*Hausner’s ratio and Carr’s Index*

Hausner’s ratio and Carr’s Index were calculated from the bulk and tapped densities of samples weighing around 3 g placed into 10-mL measuring cylinders.

The bulk density was determined from the volume occupied by the samples before tapping, while tapped density by tapping the samples until a final consistent volume was reached, which corresponds to the maximum packing density of the material. The following equations were used.

Hausner’s ratio = Tapped density/Bulk density

Carr’s index = 

[Tapped density−Bulk density]100/Tapped density

*Packability determination*

Three grams of each sample were poured slowly and gently into a 10-mL measuring cylinder and manually tapped for 100, 200, 300, 400, 500, 600,700, 800, 900,1000, 1100, and 1200 times. The packability was evaluated by the tapped density according to the Kawakita and Ludde equation as follows: (n/C (= (1/ab) + (n/a), where n is the tap number, C denotes the volume reduction which can be calculated by C = V_0_−V_n_/V_0_, where V_0_ and V_n_ are the powder bed volumes at the initial and n^th^ tapped state, respectively. The plot of n/C versus n is linear, where 1/a is the slope that indicates compactibility, and 1/ab is the intercept that indicates cohesivity (31). 

*Particle size determination*

The mean particle size of glimepiride, pure excipients and spherical agglomerates with the best flowability and packability results was determined by randomly counting the average diameter of 100 particles with an optical microscope (Leica DMLB, Germany) and their microphotographs were taken via a miniature video camera. (JVC, TK-C1380, USA).

*Physicochemical characterization of optimized spherical agglomerates*

*FT-IR spectroscopy*

FT-IR spectroscopy was used to investigate the probability of chemical interactions between the ingredients of the optimized system using infrared spectrophotometer: Shimadzu IR-435, Kyoto, Japan. The scanning was performed within a wave number of 4000 to 500 cm^−1^. 

*Differential scanning colorimetry (DSC)*

DSC was performed for glimepiride, pure excipients and the optimized SA using Differential Scanning Colorimeter: Model DSC-50; Shimadzu, Kyoto, Japan. The samples of 3-4 mg were placed in a flat-bottomed aluminum pan and heated in an atmosphere of nitrogen at a temperature range of 20-250 °C at a rate of 10 °C/min.

*X-ray powder diffraction (XRPD)*

The X-ray diffraction pattern for glimepiride, pure excipients and SA in question were recorded at room temperature using X-ray diffractometer: Model XGEN-4000, X1-advanced diffraction systems; Scintag Corp., USA at 45 kv and 40 mA current. The scanning rate was 2 °C/min over a diffraction angle (2 Ɵ°) range of 5-70 °C.

*Scanning electron microscopy (SEM)*

The shape and surface topography of glimepiride, pure excipients and optimized SA were observed through a scanning electron microscope (Joel Corp., Mikaka, Japan) operated at 15 kv after coating with gold. Different magnifications of the particles were obtained.

## Results and Discussion

*Optimization of spherical agglomeration process*

*Preliminary trials*

Several preliminary trials were made to find suitable conditions for the preparation of SA of glimepiride with good shape and yield (Table 1).

First, DCM was tried as the good solvent, water as the poor, and CHCl_3_ as the bridging liquid. Different volumes of DCM and water were used, but no drug agglomeration occurred, and only milky white emulsion droplets were formed.

Upon increasing the weight of the drug used and fixing the volumes of DCM and water, unstable emulsion droplets were formed, with no drug agglomeration. Increasing the volume of CHCl_3_ or switching to toluene (as a bridging liquid) did not affect the results.

Using CCl_4_ and benzene as bridging liquids gave more stable emulsion droplets, but no drug agglomeration occurred.

DMF was then tried as a good solvent instead of DCM, while the other variables were kept constant. Upon the addition of water, the drug started to precipitate in the form of SA. Fine droplets were observed immediately, suggesting the diffusion of the good solvent out and the poor solvent into the emulsion droplets. Using CCl_4_ or benzene as bridging liquids in this case helped bridge the drug particles together, which led to the drug agglomeration into a more perfect spherical shape.

An agitation speed of 200 rpm was then tried during the agglomeration process, but the agglomerates formed were irregular in shape. When the agitation speed of 900 rpm was tried, the agglomerates formed were split into smaller ones due to the high agitation speed. 

When the temperature of the medium was increased to 40 ºC, the agglomeration process was not efficient. This might be due to increased drug solubility in DMF at a higher temperature. 

*Physicochemical evaluation of the prepared spherical agglomerates*

*Yield percent and drug content*

*Effect of adding Aerosil in the drug solution*

After optimizing the agglomeration process, a poor yield of SA was detected. A high rate of collision and coalescence of droplets in the premature stage was accused for such poor yield. The separated solid particles adhered to the wall of the vessel and/or to the blades of the stirrer. Adding Aerosil to the drug solution before emulsion formation greatly enhanced the final yield of the product to 90% or more (Table 3). This could be reasonable, since Aerosil acted as a dispersing agent, where it could prevent the coalescence of droplets. The produced SAs were compact and did not adhere to the wall of the vessel (32). Increasing the Aerosil amount to reach the drug to-Aerosil ratio 1:2 did not further enhance the yield. This was probably due to the increased viscosity of the dispersed phase. The produced larger droplets coalesce more easily causing a drop in the final yield.

*Effect of adding hydrophilic polymers in the aqueous phase*

The addition of hydrophilic polymers assisted the role of Aerosil in increasing the yield of intact SA with a different mechanism (Table 3). These water-soluble polymers were thought to act as stabilizers for the formed emulsion. They acted by protecting the adjacent droplets from fusion and coalescence, thereby contributing to imparting sphericity and strength to the formed agglomerates (33, 34).

*Effect of adding different carriers in the drug solution*

All tested carriers were added to the drug solution at a concentration of 0.5% w/v. A further increase in concentration caused the disaggregation of particles (Table 2). These carriers were thought to modify the crystal habit of aggregates by adsorbing onto their growing surface (35). The yield of SA prepared with different polymers (namely SAPVA, SAHPC, SAPVP), carriers (namely SAps, SApf, SAst, SAac, SAgl and SAcp) or a carrier-polymer combination (having the same abbreviations as the respective carrier and polymer) ranged from 90-96%. 

The glimepiride assay in all the prepared systems showed ≈ 90% to 97% content, assuming a minimal drug loss during the dispensation procedures (Table 3).

*Kinetic analysis of the dissolution data*

The data obtained from the dissolution experiments were treated statistically according to the linear regression analysis. They were fitted to zero order, first order and Higuchi diffusion model. Kinetic treatment of data was then performed for the order of the best fit (Table 4).

*Effect of different hydrophilic polymers in the aqueous phase on the dissolution t*_1/2_

**Figure 1:**
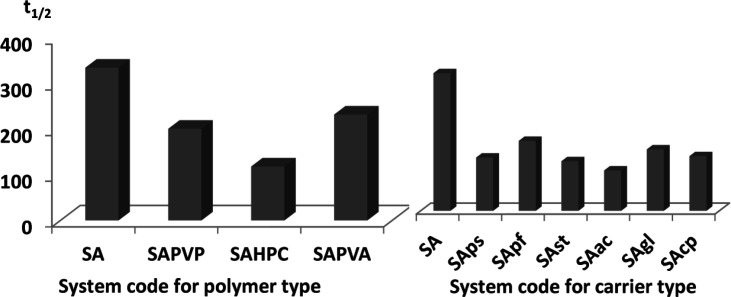
Effect of adding different polymers or carriers on the dissolution t_1/2_ of glimepiride from respective spherical agglomerates

As shown in Figure 1 and Table 4, SA alone did not greatly influence the value of t_1/2_ (334 min) when compared to the drug alone (381 min). However, introducing water soluble polymers in the aqueous phase of the emulsion caused a slight enhancement in glimepiride dissolution. Half-life values ranged from 118 min to 231 min for the three tested polymers, indicating similar efficiency in emulsion stabilization (Figure 1).

*Effect of different carriers added to the drug solution on the dissolution t*_1/2_

The introduction of carriers within the drug solution during emulsification had a great effect on dissolution enhancement. In fact, t_1/2 _dropped to more than half of the original value in case of pure drug (381 min) for all tested carriers (Figure 1) (Table 4).

For partially soluble carriers, the best enhancement was attained with Starlac followed by PreGelSt and then Pearlitol flash. The superiority of Starlac could be attributed to its spherical shape that could tailor the crystal habit of the drug to a perfect spherical geometry during the progress of agglomeration. Moreover, its adsorption on the aggregated drug crystals promoted their wetting and amorphisation.

For water-insoluble carriers, the use of Ac-Di-Sol resulted in the least recorded t_1/2 _followed by CP and then Glycolys^®^. This result could be interpreted on the basis of the strong swelling capacity of Ac-Di-Sol and CP (36, 37). Their ability to drag a large volume of the dissolution medium could strongly wet the agglomerates; thus, enabling their dissolution at a much higher rate.

*Effect of carriers in the presence of different hydrophilic polymers with respect to their effect on the dissolution t*_1/2_

**Figure 2:**
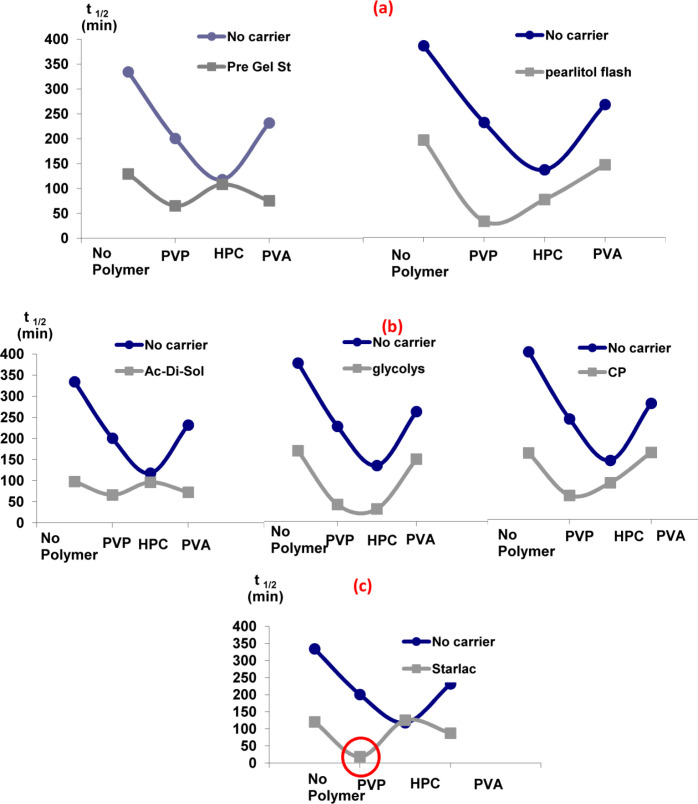
Effect of adding carriers in the drug solution in the presence of different hydrophilic polymers in the aqueous phase with respect to their effect on the dissolution t_1/2_ (a) water-soluble to partially water-soluble carriers, (b) water-insoluble carriers, (c) optimized carrier

It is obvious from Figure 2 that the addition of carriers in the drug solution along with hydrophilic polymers in the aqueous phase was the optimum choice. A sharp drop in t_1/2_ was detected for all carrier-polymer combinations relative to their analogues devoid of carriers. The probable adsorption of carriers onto the surface of SA could modify and reorganize their crystal habit to a final amorphous form (15). Therefore, it can be concluded that such synergistic combination provided a good medium for the separation of perfect spherically shaped agglomerates with a high wetting power and, accordingly, improved the dissolution rate

*Comparison of spherical agglomerates prepared with different polymers and carriers with respect to their effect on the dissolution t*_1/2_

**Figure 3:**
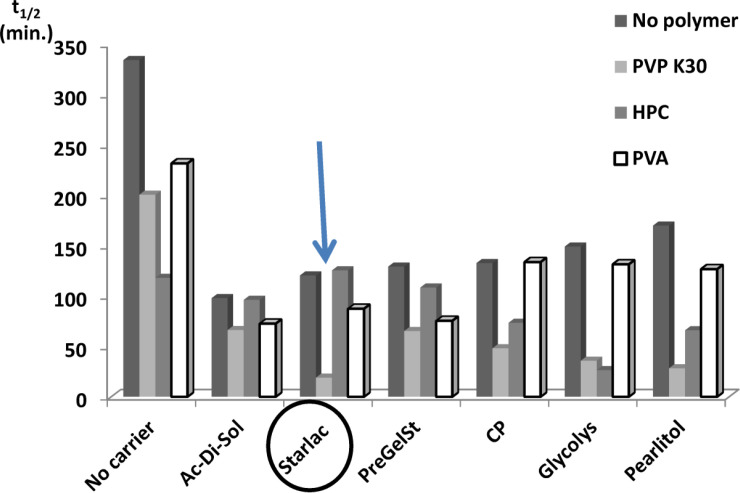
Comparison of spherical agglomerates containing different hydrophilic polymers and carriers with respect to their effect on t_1/2_

Figure 3 illustrates the superiority of SA prepared with PVP (least t_1/2_) for all tested carriers. This could be attributed to higher emulsifying and film-forming ability than other testing polymers. PVP might efficiently decrease the surface tension between phases and thus imparted a temporary physical stability to the formed quasi emulsion. In this way it contributed to the spheronization and amorphisation of the agglomerates through stabilization of the spherical droplets of the emulsion (38). Amorphous spherical particles showed higher dissolution rate than the untreated drug crystals. 

Systems with the least values for the dissolution half-life (SAPVPps, SAPVPpf, SAPVPst, SAPVPac, SAPVPgl, SAPVPcp, SAHPCpf, SAHPCgl) were considered optimum and were selected for further investigation.

*Micromeritics of optimized spherical agglomerates*

*Angle of repose*

Table 5 shows that glimepiride (with a distinct crystalline nature) exhibited very poor flowability (Ɵ ≈ 47). The angle of repose for SAPVPgl and SAPVPcp were about 28.057 and 28.854, respectively. For SAHPCgl and SAPVPst, it reached 24.781 and 24.749, respectively. All four agglomerates were considered to have excellent flowability according to the USP standards (27, 28). Spheronization and amorphisation of particles were the main reason for these results. The difference in values among different SAs could be interpreted by the variation in their surface roughness due to the different adsorbed carriers on their surfaces.

*Hausner’s ratio and Carr’s index*

It was remarkable that all prepared agglomerates possessed much lower values of tapped densities than that of the pure drug (Table 5). This might be the result of increasing the interparticulate porosity of SA after acquiring the spherical shape during agglomeration.

Hausner’s ratio and Carr’s index for pure glimepiride were 2.16 and 27%, respectively, which indicated a very poor flowability. The prepared agglomerates showed lower values, indicating the improvement of flow properties. SAPVPst possessed the least values among all tested agglomerates and could be considered as an optimized formula (29, 30). It could finally be concluded that the decrease in the values of the angle of repose, Hausner’s ratio and Carr’s index for the prepared agglomerates relative to the pure drug had a significant indication of the improved flow properties of such systems. This might be due to the large and spherical shape of agglomerates obtained. On the contrary, the fine irregular shapes of drug particles tended to have high surface-to-mass ratios than coarser particles. They might produce irregular flow properties due to differences in interparticulate contact areas.

*Packability determination*

The results obtained in Table 6 explain the packability of the prepared agglomerates when compared with that of the pure drug. The treatment of tapping was done according to Kawakita and Ludde’s equation (31). The plot of n/C versus n was linear, where 1/a is the slope that indicated compactibility and 1/ab was the intercept that indicated the cohesivity of systems. It was found that the values of the slope (1/a) for the prepared agglomerates increased with respect to the drug, indicating increased compactibility of such agglomerates, *i.e.* the agglomerates were closely packed together. As a result, the values of parameter (a) as obtained from the reciprocal of the slope of the line decreased for all the tested agglomerates.

On the other hand, the values of the intercept (1/b) decreased for the treated agglomerates with respect to the drug, indicating decreased cohesivity of such agglomerates. Accordingly, parameter (b) as obtained from the reciprocal of the intercept of the line represented the velocity of the flow of agglomerates. The values of parameter (b) obtained increased for all the tested carriers except for SAPVPac, which was slightly lower than that of the drug.

The decreased (a) values and increased (b) values suggested enhanced packability of the prepared agglomerates when compared to the pure drug. This might be explained by the large size and sphericity of the agglomerates formed. During tapping, the smaller particles went into the voids between the larger ones; hence, giving better packability to the systems (26, 39). 

SAPVPst agglomerates showed optimum flowability and packability results as compared to other systems, so they were selected for particle size determination.

*Particle size determination*

**Figure 4.**
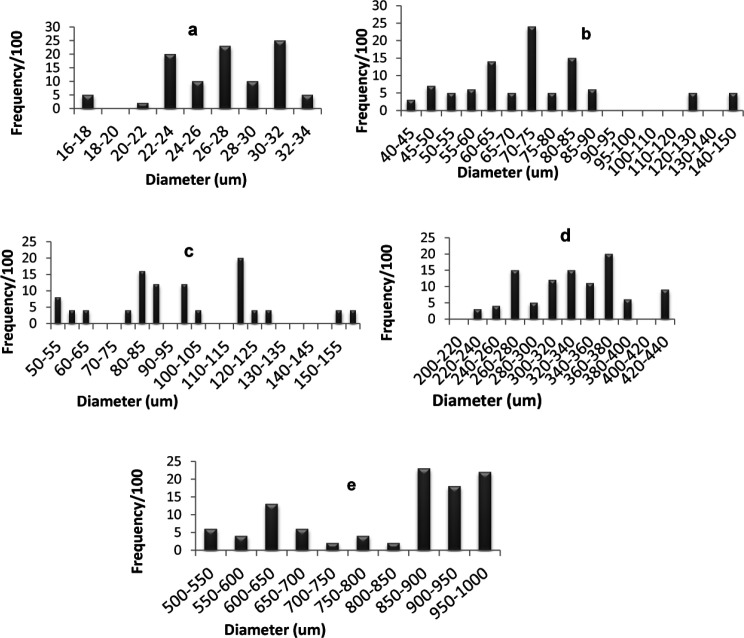
Histogram showing particle size distribution for (a) glimepiride, (b) Starlac, (c) Aerosil 200, (d) PVP K 30, (e) spherical agglomerates SAPVPst

**Figure 5:**
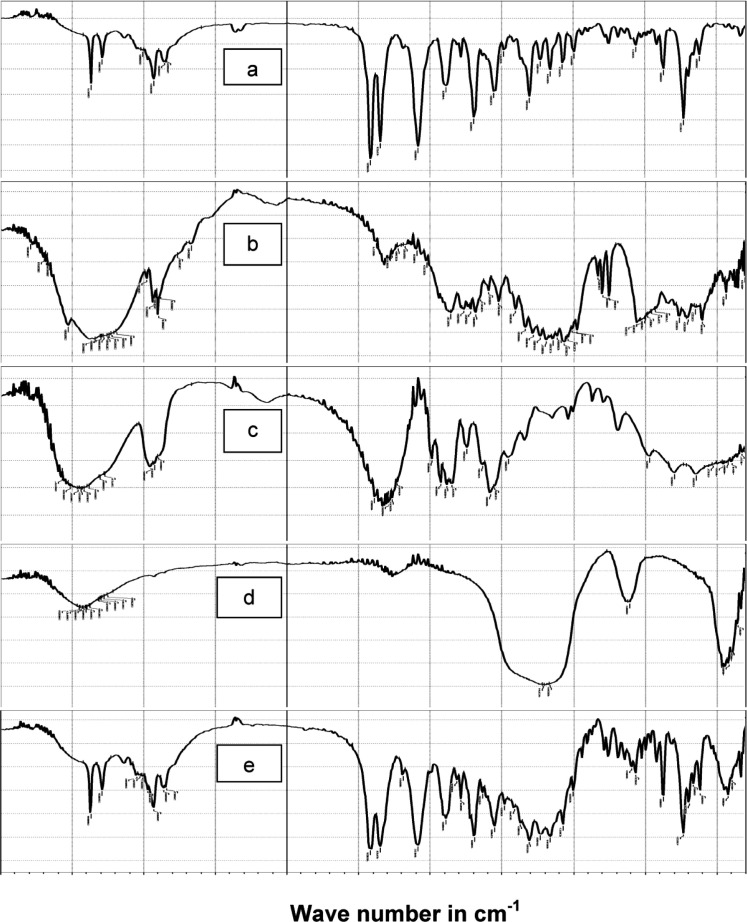
Comparison of FTIR spectra of (a) pure glimepiride, (b) Starlac, (c) PVP K30, (d) Aerosil 200, (e) SAPVPst

Figure 4 shows the percentage frequency at each particle size range for optimized SAs and the components used in their preparation. The average diameter of glimepiride particles was around 26.93 µm, Starlac about 74.92 µm, PVP K30 332.89 µm, and the formed SAPVPst agglomerates around 821.2 µm. It is, thus, obvious that the size of the prepared agglomerates was higher than all of their individual components. Furthermore a non homogeneous distribution of particles around the mean was illustrated as particles were skewed towards the large size range (850-1000 µm) (Figure 5e). 

For SAPVPst, a horizontal and a vertical diameter were measured for each particle, and their ratio was close to 1, indicating high spheronization of the formed agglomerates (40, 41).

*Physicochemical characterization of the optimized system*

It is obvious that Starlac performance was superior when PVP K30 was present in the agglomeration medium, in terms of the highest dissolution profile and the least recorded t_1/2 _reaching a minimum of 19 min. That was why further characterization was performed on the optimum agglomerates containing both Starlac and PVP K30.

*FT-IR analysis*

In order to test for possible interaction between the drug and the other components in SAPVPst, each component was studied alone for the presence of characteristic peaks and then compared with the peaks appearing in SAPVPst combining all ingredients. Figure 5 shows that: (a) Pure glimepiride displayed two peaks characteristic of N-H stretching vibration at 3367 and 3290 cm^-1^ and two bands of c=o stretching at 1708 and& 1674 cm^-1^. (b) Starlac showed several broad bands of OH stretching at 3190 to 3334 cm^-1^ and characteristic bands of CH aliphatic at 2899 and 2933 cm^-1^. (c) PVP K30 showed several broad bands of OH stretching at 3271 to 3572 cm^-1^ along with a broad band stretching of CH aliphatic at 2895, 2926, 2954 cm^-1^. A characteristic C=O stretching peak was also seen at 1643 cm^-1^. (d) Aerosil showed broad bands of OH stretching at 3375 to 3452 cm^-1^. (e) SAPVPst exhibited all the characteristic bands of the drug at the same position. Matching between the IR spectra of the pure drug and SAPVPst revealed no sign of chemical interaction either in the region of stretching vibration or in the fingerprint region.

*Differential Scanning Colorimetry (DSC)*

**Figure 6:**
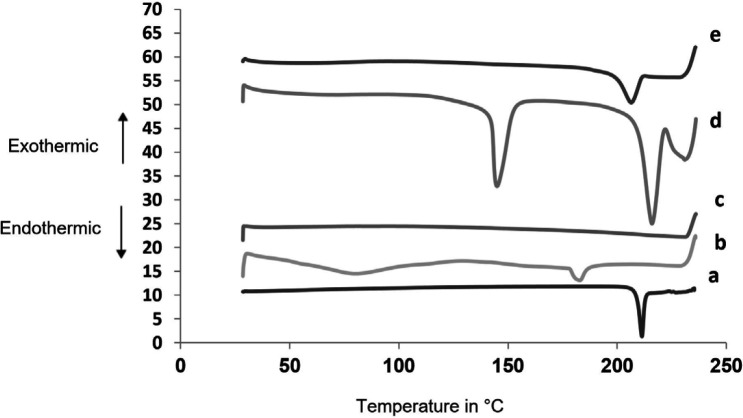
DSC thermograms for (a) pure glimepiride, (b) PVP K30, (c) Aerosil 200, (d) Starlac, (e) SAPVPst

Figure 6 shows thermograms for SAPVPst and its component excipients. Glimepiride exhibited a well defined melting peak at 215.3 °C indicating its crystalline nature. PVP K30 had two endothermic peaks at 80.23 °C and 183.2 °C. Starlac showed two characteristic endotherms at 215.83 °C and 144.89 °C. Aerosil 200 showed no sharp endotherms. The peak characteristic of SAPVPst was much less in intensity than that of glimepiride, indicating the loss of the strong crystalline nature of the drug within the SA. 

*X-ray powder diffraction*

**Figure 7:**
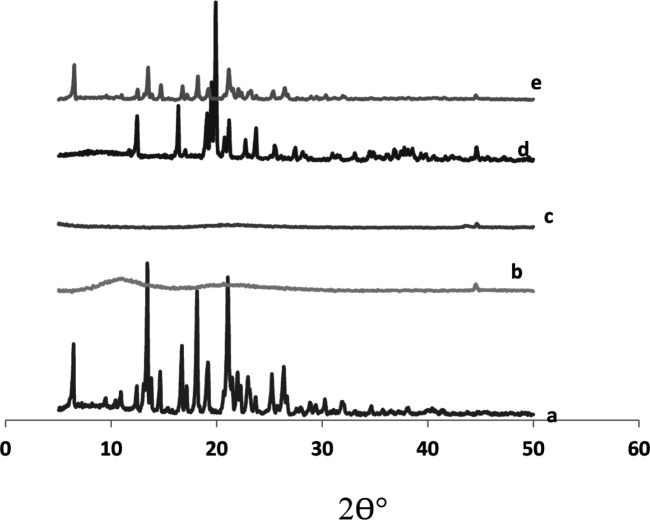
X-ray diffraction patterns of (a) pure glimepiride, (b) PVP K30, (c) Aerosil 200, (d) Starlac, (e) SAPVPst

The X-ray diffractogram of glimepiride exhibited sharp and intense peaks at 2 θ° equivalent to: 18.10°,19.12°, 22.00°, 25.21°, 26.32°, besides a series of smaller peaks at 6.35°, 14.62°, 17.02°, 22.82°, and 23.64° as shown in Figure 7. The above pattern clearly showed the strong crystal habit of the pure drug.

Starlac showed an intense peak at 2 θ° equivalent to 19.87°, besides some small peaks at 12.48°, 16.33°, 21.14°, and 23.72°. This pattern could suggest the crystalline nature of this excipient.

PVP K30 exhibited two short broad peaks at 10.98° and 20.62°, besides a small sharp one at 44.52°. Aerosil 200 showed a short broad peak at 22.03° and two short sharp peaks at 43.48° and 44.6°. The diffractogram of such excipients suggested their amorphous structures with respect to Starlac. A remarkable decrease in peak intensities characteristic of the drug were observed in the X-ray diffractogram of SAPVPst. Some peaks characteristic to Starlac were also seen, but at much lower intensity. SAPVPst were, thus, successful in converting the strong crystalline nature of the drug into an amorphous structure. 

*Scanning electron microscopy (SEM)*

**Figure 8:**
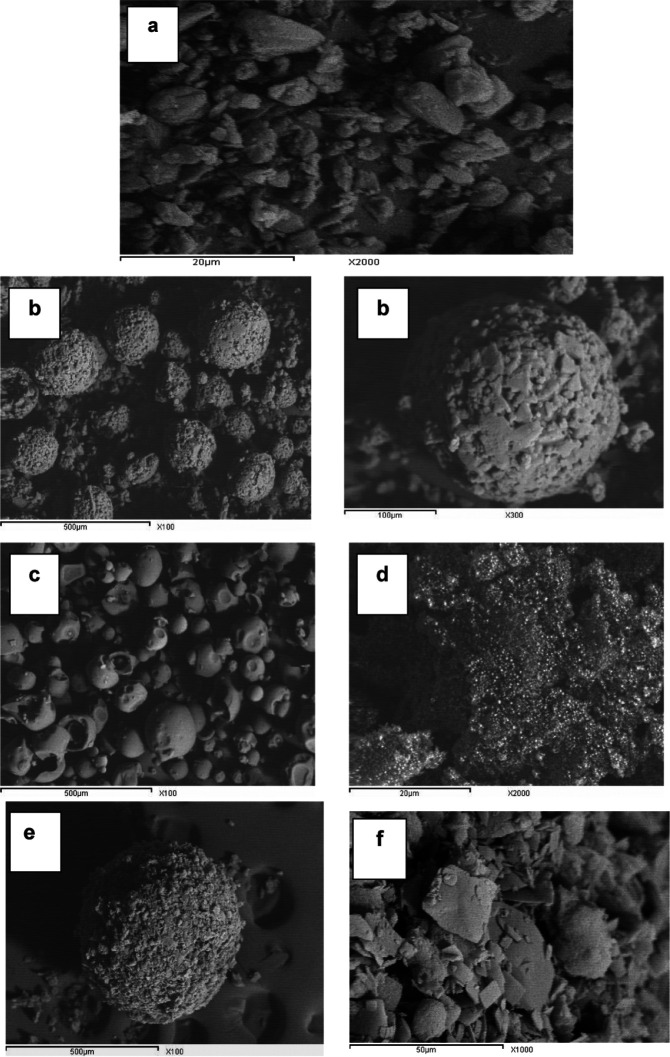
SEM of (a) pure glimepiride, (b) Starlac at 100x, 300x, (c) PVP K30 at 100x, (d) Aerosil 200 at 2000x, (e) SAPVPst whole spheres at 100x, (f) SAPVPst surface at 10000x

The surface topography of glimepiride platelets illustrates the strong crystal habit of the drug with distinct sharp edges (Figure 8); Starlac particles were globular in shape with irregular surface similar to lactose globules (which constitute the larger percentage of such carrier). PVP K30 appeared as large smooth spheres. Aerosil 200 appeared as fine particles. The prepared SAPVPst agglomerates were much larger in size compared with the single components, perfectly spherical with a distinct rough surface. Higher magnification of agglomerate surfaces showed the aggregation of drug platelets together with occasional small spherical patches that could be due to the surface adsorption of the Starlac particles.

**Table 1:** Preliminary tested variables in the optimization of spherical agglomeration process

**System** **code**	**Weight of glimepiride (mg)**	**Good solvent**		**Volume of poor solvent [water] (mL)**	**Bridging liquid**		**Agitation Speed** **(rpm)**	**Temperature** **(ºC)**
		**Type**	**Volume (mL)**		**Type**	**Volume (mL)**		
S1	50	DCM	5	10	CHCl_3_	0.25	500	25
S2	50	DCM	3	10	CHCl_3_	0.25	500	25
S3	50	DCM	2	10	CHCl_3_	0.25	500	25
S4	50	DCM	2	7	CHCl_3_	0.25	500	25
S5	50	DCM	2	5	CHCl_3_	0.25	500	25
S6	50	DCM	2	4	CHCl_3_	0.25	500	25
S7	100	DCM	2	4	CHCl_3_	0.25	500	25
S8	150	DCM	2	4	CHCl_3_	0.25	500	25
S9	150	DCM	2	4	CHCl_3_	0.5	500	25
S10	150	DCM	2	4	CHCl_3_	0.85	500	25
S11	150	DCM	2	4	Toluene	0.5	500	25
S12	150	DCM	2	4	Toluene	0.85	500	25
S13	150	DCM	2	4	CCl_4_	0.5	500	25
S14	150	DCM	2	4	CCl_4_	0.85	500	25
S15	150	DCM	2	4	Benzene	0.5	500	25
S16	150	DCM	2	4	Benzene	0.85	500	25
S17	150	DMF	2	4	CCl_4_	0.5	500	25
S18	150	DMF	2	4	CCl_4_	0.85	500	25
S19	150	DMF	2	4	Benzene	0.5	500	25
S20	150	DMF	2	4	Benzene	0.85	500	25
S21	150	DMF	2	4	Benzene	0.85	200	25
S22	150	DMF	2	4	Benzene	0.85	900	25
S23	150	DMF	2	4	Benzene	0.85	500	40
^***^S24	150	DMF	2	4	Benzene	0.85	500	40

DCM: Dichloromethane (Methylene chloride); DMF: Dimethylformamide; CHCl_3_: Chloroform; CCl_4: _Carbon tetrachloride; ^***^S24: Glimepiride was added to a mixture of DMF and benzene then the mixture poured into water.

**Table 2:** Tested variables in optimization of spherical agglomeration with added excipients

**System code**	**Poor Solvent **		**Excipients added to the drug solution **		
	**Additive**	**Volume ** **(mL)**	**Aerosil 200 ** **(mg)**	**Carrier**	
				**Type**	** w/v (%)**
SA	-	4	150	-	-
SA"	-	4	300	-	-
SAPVA	PVA	4	150	-	-
SAHPC	HPC	4	150	-	-
SAPVP	PVP K30	4	150	-	-
SAps	-	4	150	PreGelSt	0.5
SApf	-	4	150	Pearlitol flash	0.5
SAst	-	4	150	Starlac	0.5
SAac	-	4	150	Ac-Di-Sol	0.5
SAgl	-	4	150	Glycolys^®^	0.5
SAcp	-	4	150	CP	0.5
SAPVAps	PVA	4	150	PreGelSt	0.5
SAPVAps"	PVA	4	150	PreGelSt	0.75
SAPVApf	PVA	4	150	Pearlitol flash	0.5
SAPVAst	PVA	4	150	Starlac	0.5
SAPVAac	PVA	4	150	Ac-Di-Sol	0.5
SAPVAgl	PVA	4	150	Glycolys^®^	0.5
SAPVAcp	PVA	4	150	CP	0.5
SAHPCps	HPC	4	150	PreGelSt	0.5
SAHPCpf	HPC	4	150	Pearlitol flash	0.5
SAHPCst	HPC	4	150	Starlac	0.5
SAHPCac	HPC	4	150	Ac-Di-Sol	0.5
SAHPCgl	HPC	4	150	Glycolys^®^	0.5
SAHPCcp	HPC	4	150	CP	0.5
SAPVPps	PVP K30	4	150	PreGelSt	0.5
SAPVPpf	PVP K30	4	150	Pearlitol flash	0.5
SAPVPst	PVP K30	4	150	Starlac	0.5
SAPVPac	PVP K30	4	150	Ac-Di-Sol	0.5
SAPVPgl	PVP K30	4	150	Glycolys^®^	0.5
SAPVPcp	PVP K30	4	150	CP	0.5

All spherical agglomerates were prepared at an agitation speed of 500 rpm and at a temperature of 25 ºC. They contained Glimepiride weight = 150 mg, good solvent = 2 mL DMF, bridging liquid = 0.85 mL CCl_4_ and a poor solvent = 4 mL.

**Table 3:** Glimepiride content and yield percent of spherical agglomerates

**System Code**	**Drug Content ** **(w/w%) ± SD**	**Yield** **(w/w%) ± SD**
SA	92.07 ± 1.05	90.23 ± 1.77
SAPVP	92.83 ± 1.17	92.14 ± 2.09
SAHPC	93.63 ± 1.2	89.54 ± 1.51
SAPVA	93.64 ± 1.13	90.96 ± 1.04
SAps	91.16 ± 2.54	93.65 ± 1.72
SAPVPps	92.54 ± 1.55	95.27 ± 1.9
SAHPCps	93.36 ± 1.84	90.01 ± 1.11
SAPVAps	95.67 ± 1.68	91.36 ± 2.56
SApf	92.73 ± 1.83	95.25 ± 2.45
SAPVPpf	93.64 ± 1.5	93.87 ± 1.75
SAHPCpf	92.04 ± 1.18	95.21 ± 2.33
SAPVApf	93.56 ± 1.34	91.47 ± 1.48
SAst	93.02 ± 1.98	95.37 ± 2.24
SAPVPst	92.20 ± 1.8	92.89 ± 1.7
SAHPCst	93.87 ± 1.33	89.99 ± 1.84
SAPVAst	94.91 ± 1.28	91.08 ± 1.1
SAac	93.49 ± 1.38	90.56 ± 1.44
SAPVPac	93.87 ± 1.26	96.58 ± 1.61
SAHPCac	94.25 ± 1.75	92.07 ± 2.83
SAPVAac	94.59 ± 1.09	90.43 ± 1.2
SAgl	92.92 ± 1.21	94.21 ± 2.02
SAPVPgl	94.31 ± 1.56	92.00 ± 1.18
SAHPCgl	90.02 ± 1.62	96.78 ± 1.74
SAPVAgl	94.39 ± 1.89	94.24 ± 2.09
SAcp	96.90 ± 1.1	90.23 ± 1.62
SAPVPcp	92.49 ± 1.54	92.47 ± 1.12
SAHPCcp	95.13 ± 1.6	94.27 ± 1.7
SAPVAcp	88.98 ± 1.09	89.99 ± 2.01

SD: Standard deviation.

**Table 4.** Kinetic treatment of dissolution data of glimepiride from spherical agglomerates

**System** **Code**	**Order of Release**	**K** ^#^	**Half-life (min)**
SA	first	2.0727× 10^-3^	334.34
SAPVP	first	3.4545×10^-3^	200.60
SAHPC	zero	0.16	118.00
SAPVA	first	2.99 ×10^-3^	231.77
SAps	zero	0.18	129.29
SAPVPps	zero	0.25	65.24
SAHPCps	diffusion	3.41	108.44
SAPVAps	diffusion	2.84	75.45
SApf	zero	0.12	169.92
SAPVPpf	zero	0.26	28.24
SAHPCpf	first	0.01	66.00
SAPVApf	diffusion	3.49	126.80
SAac	diffusion	3.36	97.92
SAPVPac	zero	0.21	66.26
SAHPCac	diffusion	2.55	96.26
SAPVAac	zero	0.21	72.64
SAgl	zero	0.16	149.11
SAPVPgl	zero	0.23	35.73
SAHPCgl	diffusion	4.47	26.49
SAPVAgl	zero	0.19	131.52
SAcp	zero	0.19	132.74
SAPVPcp	zero	0.21	48.17
SAHPCcp	zero	0.23	73.44
SAPVAcp	zero	0.19	133.74
SAst	zero	0.18	120.43
SAPVPst	diffusion	6.27	18.92
SAHPCst	first	5.52× 10^-3^	125.54
SAPVAst	zero	0.29	87.41
Drug	zero	0.08	381.19

^#^K in mg/min in zero order, min^-1 ^in first order and in mg/min^1/2^ in Higuchi model.

**Table 5:** Powder flow measurements for optimized spherical agglomerates

**System** **Code**	**Bulk Density (g/cm** ^3^ **)**	**Tapped Density (g/cm** ^3^ **)**	**Angle of repose**		**Hausner's Ratio**		**Carr's Index**	
			**Value (º)**	**Flow Indication**	**Value**	**Flow Indication**	**(%)**	**Flow Indication**
Pure glimepiride	0.375	0.811	47.124	Poor	2.162	V.V.poor	27.027	Poor
SAPVPps	0.322	0.361	31.591	Good	1.121	Good	12.048	Good
SAPVPpf	0.341	0.411	35.251	Good	1.205	Fair	13.698	Good
SAPVPst	0.312	0.319	24.749	Excellent	1.021	Excellent	10.638	Excellent
SAPVPac	0.337	0.428	39.941	Fair	1.271	Passable	21.348	Passable
SAPVPgl	0.315	0.344	28.057	Excellent	1.091	Excellent	11.494	Excellent
SAPVPcp	0.326	0.357	28.854	Excellent	1.095	Excellent	11.904	Excellent
SAHPCpf	0.315	0.375	32.415	Good	1.187	Good	15.789	Good
SAHPCgl	0.322	0.348	24.781	Excellent	1.081	Excellent	11.627	Excellent

**Table 6:** Packability parameters for optimized spherical agglomerates

**System**	**(a)-Value** **compactibility constant**	**(b)-Value** **velocity** **constant **
Pure glimepiride	1.561	0.000521
SAPVPps	0.275	0.001411
SAPVPpf	0.199	0.000833
SAPVPst	0.041	0.001181
SAPVPac	0.625	0.000502
SAPVPgl	0.156	0.001111
SAPVPcp	0.237	0.000801
SAHPCpf	0.493	0.000526
SAHPCgl	0.111	0.001681

## Conclusion

 The enhanced dissolution rate of glimepiride from optimized spherical agglomerates supports the agreement that the new quasi-emulsion, crystallo-co-agglomeration method was successful in promoting enhanced wetting ability, amorphisation and spheronization of the final product. This situation is brought about due to a prime effect of an adsorbed starch based carrier onto the surface of the final spherical particles along with the hydrophilic polymer added in the aqueous phase of the emulsion.
